# Effects of the Freezing Diluent Tonicity and Prefreezing Cooling Rates on Post‐Thaw Sperm Quality Markers in the Pure‐Bred Arabian Stallion

**DOI:** 10.1155/vmi/4438005

**Published:** 2026-08-13

**Authors:** Leili Mavalizadeh, Mohammad Reza Divar, Asghar Mogheiseh, Maryam Emami

**Affiliations:** ^1^ School of Veterinary Medicine, Shiraz University, Shiraz, Fars, Iran, shirazu.ac.ir; ^2^ Department of Clinical Sciences, School of Veterinary Medicine, Shiraz University, Shiraz, Fars, Iran, shirazu.ac.ir; ^3^ Baghaee’s Arabian Horse Stud, Abadeh, Fars, Iran

**Keywords:** freezing, prefreezing cooling rates, sperm, stallion, tonicity

## Abstract

Stallion semen cryopreservation remains limited by inconsistent post‐thaw sperm quality and fertility. This study evaluated the effects of freezing extender effective tonicity and prefreezing cooling rate on post‐thaw sperm characteristics in pure‐bred Arabian stallions. In a two‐phase design, ejaculates (*n* = 40) from 10 stallions were processed using extenders with different osmotic conditions (330, 390, and 450 mOsm/kg) and subjected to cooling durations of 45 min, 1.5 h, or 3 h. Sperm quality was assessed using CASA, functional assays, flow cytometry, transcriptomic analysis, and a pilot fertility trial. Moderate hypertonicity (∼356 mOsm/kg effective tonicity) significantly improved sperm function, including higher proportions of live/nonapoptotic cells, enhanced mitochondrial activity, improved acrosome integrity, and reduced DNA fragmentation (*p* < 0.05). In contrast, excessive hypertonicity (∼383 mOsm/kg) increased necrosis and acrosome damage and tended to elevate the Bax/Bcl2 ratio although this effect was not statistically significant. Sperm kinematic parameters were not affected by osmolarity. Extending prefreezing cooling from 45 min to 1.5–3 h significantly improved motility, viability, mitochondrial function, acrosome stability, and DNA integrity, while reducing apoptosis and total cell death (*p* < 0.05), with no consistent additional benefit beyond 1.5 h. Oxidative stress (MDA) was not significantly affected. The optimized protocol resulted in pregnancy rates of 75.0% at Day 21 and 62.5% at Day 60. In conclusion, moderate hypertonicity combined with controlled slow cooling enhances stallion sperm cryosurvival and provides a practical strategy to improve fertility outcomes.

## 1. Introduction

Semen cryopreservation and artificial insemination (AI) are integral components of modern equine breeding, enabling long‐term preservation of genetic material, global dissemination of superior stallion lines, and mitigation of disease transmission risks [1, 2]. Despite these advantages, the routine use of frozen semen in horses remains limited compared with other domestic species [3], primarily due to the inherent sensitivity of stallion spermatozoa to cryo‐induced damage compared with other domestic species and the resulting variability in post‐thaw fertility outcomes [4]. Improving the efficiency and reliability of stallion semen cryopreservation, therefore, remains a critical objective in both research and clinical practice.

Extensive research efforts have focused on enhancing sperm cryosurvival through optimization of extender composition and the use of antioxidants and cryoprotective agents [1]. Beyond these biochemical approaches, increasing attention has been directed toward the physicochemical environment during cryopreservation [1], particularly the osmotic characteristics of the freezing medium and the rate of prefreezing cooling, as key determinants of sperm structural and functional integrity.

The osmotic environment plays a central role in regulating sperm cell volume, membrane stability, and intracellular ice formation during freezing. Conventional stallion semen extenders are typically formulated to be iso‐osmotic (∼330 mOsmol/kg), reflecting the osmolarity of seminal plasma [5]. However, a growing body of evidence suggests that slightly hypertonic conditions—often achieved through the addition of nonpermeable sugars such as sucrose or trehalose—can improve post‐thaw sperm survival, acrosome integrity, and mitochondrial function [2, 6–8]. These sugars are believed to exert their protective effects by inducing controlled cellular dehydration prior to freezing, thereby reducing intracellular ice formation and stabilizing membrane phospholipids [7, 9]. Nevertheless, the beneficial effects of increased osmotic pressure appear to be confined to a narrow range, as excessive hypertonicity may promote oxidative stress, membrane destabilization, and apoptosis.

In parallel, the rate at which spermatozoa are cooled from physiological to suprazero temperatures represents another critical determinant of cryosurvival. During the prefreezing cooling phase, particularly between 15°C and 5°C, sperm membranes undergo phase transitions that render them highly susceptible to cold shock and associated structural damage [10]. Suboptimal cooling rates can disrupt membrane organization, impair mitochondrial activity, and increase oxidative stress, ultimately compromising post‐thaw viability and fertilizing capacity [4, 11]. Although programmable freezers enable precise control of cooling kinetics, their cost and limited availability restrict their practical use in many clinical and field settings, underscoring the need for effective and reproducible manual cooling strategies [12].

Despite the recognized importance of both osmotic conditions and cooling dynamics, several critical limitations persist in the existing literature. First, many studies evaluating sugar supplementation have not directly measured or controlled the effective osmotic pressure (tonicity) experienced by spermatozoa, making it difficult to distinguish between osmotic and molecular protective effects. Second, osmotic conditions and cooling rates are often investigated independently, despite their likely interactive influence on cryoinjury mechanisms. Third, there is a lack of standardized, field‐applicable protocols that integrate these parameters into a practical cryopreservation strategy. Finally, the extent to which improvements in post‐thaw sperm quality translate into in vivo fertility outcomes remains insufficiently addressed.

Therefore, the present study was designed to systematically evaluate the effects of controlled effective osmotic pressure (tonicity) of the freezing extender and prefreezing cooling rates on a comprehensive set of post‐thaw sperm quality parameters in pure‐bred Arabian stallions. By implementing a two‐step dilution approach to regulate osmotic exposure and a fully manual cooling system, this study aimed to develop a robust and practical cryopreservation protocol suitable for field conditions. Furthermore, a preliminary fertility trial was conducted as a proof‐of‐concept to determine whether optimization of these physicochemical parameters translates into acceptable in vivo reproductive performance.

## 2. Materials and Methods

### 2.1. Animals and Ethics

The present investigation was conducted at an Arabian horse breeding facility in Abadeh, Fars Province, Iran, which housed 16 purebred stallions and 250 broodmares, during the breeding season from March to August 2021. Of these stallions, 10 clinically healthy adult Arabian stallions aged 7–22 years were selected based on the following stallion‐level inclusion criteria: normal general and reproductive health, no detectable reproductive tract abnormalities, a body condition score within the acceptable range, regular participation in the farm semen‐freezing program for at least two months before the study, and documented previous farm breeding records and successful reproductive use. Stallions had a mean body condition score of 5, ranging from 4 to 6, on a 9‐point scale, assessed according to the Henneke scoring system [13]. At the ejaculate level, only gel‐free semen samples without urine or blood contamination and with subjective total motility (TM) ≥ 80% were included in the study. The horses were provided with a nutritionally balanced diet consisting of alfalfa hay and wheat straw as roughage, while corn, barley, and wheat bran served as energy and protein concentrate sources, supplemented with adequate vitamins and minerals. Feeding occurred five times daily, and the horses were maintained under a controlled environment with an 18:6‐h light:dark cycle and a stable temperature of 25°C within a closed housing system. Deworming protocols adhered to established antiparasitic treatment guidelines. Compliance with the ARRIVE guidelines 2.0, the UK Animals (Scientific Procedures) Act of 1986, and EU Directive 2010/63/EU for animal experimentation was ensured. The study was approved under the Iranian laboratory animal ethics framework, under the oversight of the Iranian Society for the Prevention of Cruelty to Animals and the Shiraz University Research Council (IACUC no. 4687/63).

### 2.2. Experimental Design

**FIGURE 1:**
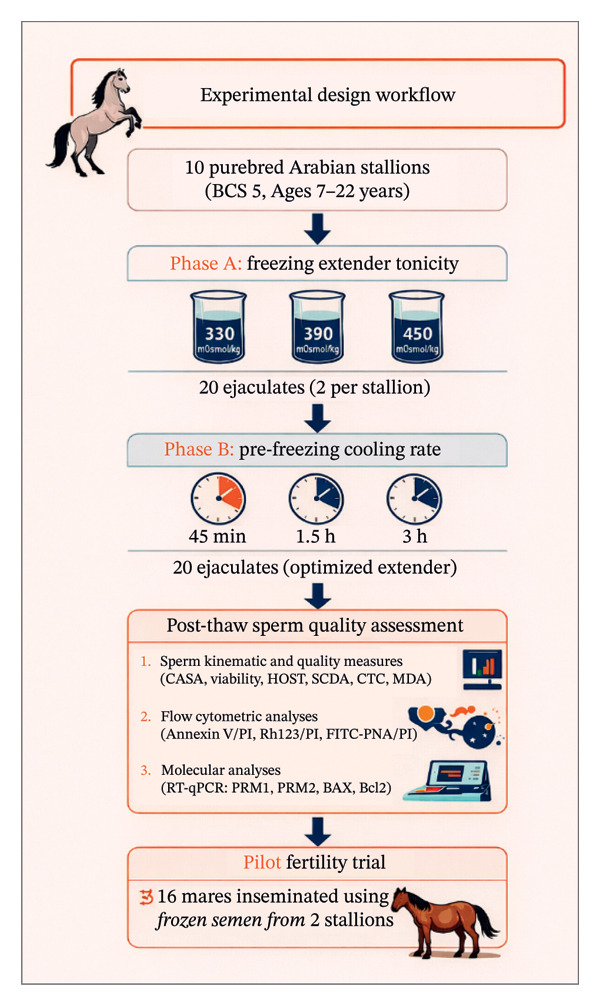
Experimental design workflow. The study comprised two sequential phases evaluating freezing extender tonicity and prefreezing cooling rates, followed by post‐thaw assessment of sperm kinematic and quality measures, flow cytometric parameters, and molecular profiles. A pilot fertility trial was conducted using semen processed under optimized conditions.

The present investigation was conducted in two distinct phases (Figure 1). In the initial phase (A), 20 semen samples obtained from 10 stallions (with two samples per stallion) were analyzed to determine the impact of the effective osmotic pressure of the freezing diluent on the parameters of post‐thaw spermatozoa. The subsequent phase (B) involved another set of 20 semen samples from the same stallions (again, two samples from each) to evaluate the influence of prefreezing cooling rates on the parameters of post‐thaw spermatozoa. The semen extenders utilized in the second phase were formulated based on the optimal results derived from the first phase of the investigation. In addition, a preliminary fertility trial was conducted using only semen samples cryopreserved under the optimal combination of osmotic pressure and prefreezing cooling rate identified in phases A and B. This phase was not designed as a comparative study among experimental groups, but rather as a validation step to assess the fertilizing capacity of sperm processed under the optimized protocol.

### 2.3. Semen Collection and Inclusion Criteria

Semen samples were collected using a Missouri‐type artificial vagina (Minitube, Germany), fitted with a commercial semen filter to separate the gel‐free fraction. Before collection, stallions were handled according to the routine farm semen‐collection protocol, and semen was collected into a prewarmed collection bottle. Immediately after collection, the gel‐free fraction was evaluated macroscopically for color and contamination, and a wet smear was prepared for subjective assessment of total sperm motility under a bright‐field microscope at × 400 magnification.

### 2.4. Preparation of Centrifugation and Freezing Extenders

The compositions and physicochemical characteristics of the centrifugation and freezing extenders are presented in Table 1. A skim milk–based centrifugation extender was prepared and used as the base medium for all treatments. The freezing extenders were formulated by supplementing the base medium with different concentrations of sucrose to achieve osmotic pressures of 330, 390, and 450 mOsmol/kg, while maintaining a constant pH of 7.2. In addition, glycerol (5%, v/v), dealbuminated fresh chicken egg yolk (5%, v/v), and sodium dodecyl sulfate (0.03%, w/v) were incorporated exclusively into the freezing extenders. All extenders were equilibrated at 37°C and sonicated for 15 min prior to use.

**TABLE 1:** Composition and physicochemical properties of the centrifugation and freezing extenders used in the study.

Component	Amount (per 50 mL)	Centrifugation extender	Freezing extender (330 mOsmol/kg)	Freezing extender (390 mOsmol/kg)	Freezing extender (450 mOsmol/kg)
Nonfat powdered milk	1.4 g	✔	✔	✔	✔
D‐(+)‐Glucose monohydrate	1.325 g	✔	✔	✔	✔
Sucrose	0.87 g	✔	✔	—	—
Sucrose	1.725 g	—	—	✔	—
Sucrose	2.585 g	—	—	—	✔
Penicillin G sodium	0.05 g	✔	✔	✔	✔
Dihydrostreptomycin sulfate	0.075 g	✔	✔	✔	✔
Sodium bicarbonate (7.5% solution)	300 µL	✔	✔	✔	✔
Glycerol (v/v)	5%	—	✔	✔	✔
Dealbuminated fresh chicken egg yolk (v/v)	5%	—	✔	✔	✔
Sodium dodecyl sulfate (SDS, w/v)	0.03%	—	✔	✔	✔
**Final pH**		**7.2**	**7.2**	**7.2**	**7.2**
**Final osmolarity (mOsmol/kg)**		**330**	**330**	**390**	**450**

*Note:* The centrifugation extender served as the base medium for all treatments, while freezing extenders were prepared by supplementing the base formulation with varying sucrose concentrations to achieve different osmotic pressures. Glycerol, egg yolk, and sodium dodecyl sulfate were included only in freezing extenders. Some values are presented in bold to improve visual contrast and enhance readability.

### 2.5. Semen Processing for Freezing

**FIGURE 2:**
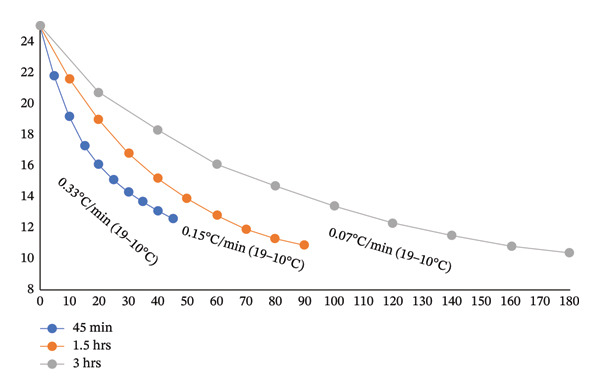
Cooling curves (25°C–10°C) applied in the present study, with mean cooling rates (°C/min), including values between 19°C and 10°C for each curve.

Upon collection, the volume and color of the ejaculates were promptly documented and subsequently diluted at a 1:1 (v/v) ratio with a prewarmed centrifugation extender maintained at 37°C. The diluted semen samples were then transported to the local farm laboratory within five minutes, also at 37°C, where they underwent centrifugation at 1000 × *g* for 20 min using a swing‐out rotor, in 50‐mL sterile plastic tubes containing 3 mL of prewarmed cushion fluid (Minitube, Germany). Following centrifugation, 95% of the supernatants and the cushion base were discarded, and the sperm density of the remaining pellet was assessed using a standard hemocytometer (Neubauer, Germany). Sufficient volumes of a washing extender (25°C, with an osmolarity of 330 mOsmol/kg water) were then added to adjust the sperm density to a final concentration of 400 million/mL. Subsequently, freezing extenders from various osmolarity groups (25°C) were incorporated at a 1:1 ratio (v/v) with the initial portion of the diluted samples to achieve a final sperm density of 200 million/mL. The diluted semen samples were then placed in a beaker containing 450 mL of 25°C tap water and cooled gradually in a standard refrigerator (5°C) over a period of 3 hours. In the second phase of the study, the diluted semen samples were cooled in beakers with varying volumes of 25°C water to manipulate the cooling rates, utilizing beakers with 450, 150, and 50 mL of water for cooling durations of three hours, 1.5 h, and 45 min, respectively (Figure 2). Once the samples reached 5°C, they were transferred into precooled 0.5‐mL plastic semen straws (Minitube, Germany) and sealed with polyvinyl chloride powder. Finally, the filled semen straws were exposed to liquid nitrogen vapor at a height of 4 cm above the liquid surface for 20 min before being immersed in liquid nitrogen. All frozen semen straws were stored in LN tanks (MVE, USA) for a minimum of 1 month prior to post‐thaw analyses. The semen straws were thawed in a 37°C water bath for 30 s.

### 2.6. Post‐Thaw Semen Assessments

Standard sperm quality assays were performed according to previously described methods with minor modifications, as cited in the following; therefore, only essential procedural details are provided. For each experimental group, at least three frozen semen straws were thawed, pooled, and evaluated after approximately 5 min of incubation at 37°C.

#### 2.6.1. Non‐Flowcytometric Evaluations

##### 2.6.1.1. Computer‐Assisted Sperm Motion Analysis

Post‐thaw sperm kinematics were evaluated using a computer‐assisted semen analysis system (CASA; Houshmand Fanavar, Tehran, Iran) connected to a bright‐field microscope with a heated chamber maintained at 37°C. For each sample, three randomly selected microscopic fields were analyzed, with at least 800 spermatozoa evaluated. The recorded parameters included total motility (TM, %), progressive motility (PM, %), curvilinear velocity (VCL, μm/s), straight‐line velocity (VSL, μm/s), average‐path velocity (VAP, μm/s), linearity (LIN, %), wobble (WOB, %), straightness (STR, %), mean angular displacement (MAD, °), amplitude of lateral head displacement (ALH, μm), and beat‐cross frequency (BCF, Hz). The CASA settings are provided in Supporting Table 1.

##### 2.6.1.2. Viability and Morphology

Sperm viability and morphology were assessed using Eosin–Nigrosin supravital staining according to standard procedures [14]. Briefly, stained smears were examined under a bright‐field microscope at × 1000 magnification, and 200 spermatozoa were evaluated per sample. Unstained spermatozoa were considered viable, whereas pink‐stained spermatozoa were considered nonviable. Sperm morphology was classified as normal or abnormal.

##### 2.6.1.3. Plasma Membrane Functional Integrity

Plasma membrane functional integrity was evaluated using the hypo‐osmotic swelling test (HOST), as previously described [15]. Briefly, semen aliquots were incubated with prewarmed hypo‐osmotic solution at 37°C for 30 min. A wet mount was then examined under a bright‐field microscope at × 400 magnification, and spermatozoa showing tail swelling or coiling were considered HOST‐positive.

##### 2.6.1.4. DNA Integrity

Sperm DNA integrity was assessed using the sperm chromatin dispersion assay (SCDA), as previously described [16]. Briefly, semen samples were embedded in low‐melting‐point agarose on precoated slides, subjected to acid denaturation and lysis, dehydrated through graded ethanol, and stained using Diff‐Quick. A total of 200 spermatozoa were evaluated under a bright‐field microscope at × 1000 magnification. Spermatozoa with large halos were classified as having intact DNA, whereas those with small or absent halos were classified as having fragmented or degraded DNA, respectively.

##### 2.6.1.5. Capacitation Status and Acrosomal Integrity

Sperm capacitation status and acrosomal integrity were assessed using the chlortetracycline fluorescence assay, as previously described [15]. Briefly, semen samples were mixed with CTC staining solution, fixed with glutaraldehyde, and examined under a fluorescence microscope at × 1000 magnification. At least 200 spermatozoa were evaluated per slide and classified as noncapacitated with intact acrosome, capacitated, or acrosome‐reacted/damaged according to their fluorescence patterns.

##### 2.6.1.6. Membrane Lipid Peroxidation (Malondialdehyde [MDA] Levels)

Lipid peroxidation was assessed by measuring MDA levels using a thiobarbituric acid–based commercial kit (ZellBio, Germany), according to the manufacturer’s instructions and a previously described method [17]. Semen samples containing 5 × 10^6^ spermatozoa were lysed by repeated freeze–thaw cycles, reacted with thiobarbituric acid under high‐temperature acidic conditions, and centrifuged. The absorbance of the supernatant was measured at 550 nm using an ELISA plate reader, and MDA concentrations were calculated from standard values. The intra‐ and interassay coefficients of variation were 5.8% and 7.6%, respectively.

#### 2.6.2. Flowcytometric Evaluations

##### 2.6.2.1. Flow Cytometer Machine Characteristics and Technical Settings

Flow‐cytometric analyses were performed using a MACSQuant® Analyzer 10 flow cytometer (Miltenyi Biotec, Germany) equipped with a 488‐nm excitation laser. Green fluorescence was detected using the B1 channel, and red fluorescence was detected using the B3 channel. Sperm populations were gated based on forward‐ and side‐scatter characteristics using unstained controls, and thresholds for positive and negative populations were established using single‐stained controls. For each sample, 10,000 events were recorded.

##### 2.6.2.2. Annexin V/Propidium Iodide Apoptosis Assay

Apoptosis‐like changes were evaluated using an Annexin V‐FITC/Propidium Iodide kit (ApoFlowEx® FITC Kit, Exbio, Czech Republic), as previously described [18]. Briefly, pooled thawed semen samples were diluted in Annexin V binding buffer, stained with Annexin V‐FITC and PI, incubated at room temperature in the dark, and analyzed immediately by flow cytometry. Spermatozoa were classified as live/nonapoptotic, early apoptotic, late apoptotic, or necrotic according to Annexin V and PI staining patterns.

##### 2.6.2.3. Rhodamine 123/Propidium Iodide Staining Assay

Mitochondrial membrane potential was assessed using Rhodamine 123/Propidium Iodide staining, as previously described [19]. Briefly, thawed semen samples were diluted in prewarmed PBS, stained with Rhodamine 123, incubated at room temperature in the dark, counterstained with PI, and analyzed immediately by flow cytometry. Spermatozoa were classified according to mitochondrial activity and membrane integrity based on Rhodamine 123 and PI fluorescence.

##### 2.6.2.4. FITC‐PNA/Propidium Iodide Staining Assay

Acrosome integrity was evaluated using FITC‐PNA/Propidium Iodide staining, as previously described [20]. Briefly, thawed semen samples were diluted in prewarmed PBS, stained with FITC‐PNA, incubated at room temperature in the dark, counterstained with PI, and analyzed immediately by flow cytometry. Spermatozoa were classified according to the acrosomal status and membrane integrity based on FITC‐PNA and PI fluorescence.

#### 2.6.3. Transcriptomic Assays

##### 2.6.3.1. Total RNA Extraction and Complementary DNA (cDNA) Synthesis

For each experimental group, three frozen–thawed semen straws were pooled for transcriptomic analysis. Total RNA was extracted from approximately 100 × 10^6^ spermatozoa using a combined TRIzol and spin‐column method according to the manufacturer’s instructions and previous recommendations for sperm RNA processing [21]. RNA quantity and purity were assessed using a NanoDrop™ spectrophotometer. cDNA was synthesized from 600 to 700 ng of total RNA using a commercial cDNA synthesis kit with oligo‐dT and random hexamer primers, and cDNA samples were stored at −20°C until analysis.

##### 2.6.3.2. PCR Primer Designing, Optimization, and Amplification Efficiency Calculation

Primer pairs were designed using the NCBI Primer‐BLAST tool and, where possible, were selected to span exon–exon junctions to minimize genomic DNA amplification. Primer specificity was confirmed in silico, and primer concentrations were optimized using SYBR Green real‐time RT‐PCR. Amplification efficiency was determined using serially diluted pooled cDNA and calculated from standard curves. Primer sequences, amplicon sizes, optimized concentrations, and amplification efficiencies are presented in Table 2.

**TABLE 2:** Primer sequences and PCR conditions used for gene expression analysis.

Gene	Forward primer (5′ ⟶ 3′)	Reverse primer (5′ ⟶ 3′)	Accession number	Exon junction	Amplicon size (bp)	Primer concentration (nM)
PRM1	GCCAGAGCCAGAGCAGAT	CGGCAGCACACTCTCCT	NM_001083596.1	Exon 106/107 (R)	—	400/600
PRM2	GCATCGCAGAGAAGGGTGA	CTTGTGGGCTCCTTGCAAAC	XM_005598914.3	Exon 392/393 (F)	—	200/200
GAPDH	CCTAACGTGTCAGTCGTGGA	AATCGCAGGAGACAACCTGG	NM_001163856.1	Exon 888/889 (R)	—	600/600
BAX	TGGAGCTGCAGAGGATGA	AGCCCATGATGGTCCTGA	XM_023650076.1	Exon 732/733 (F)	—	600/200
Bcl‐2	GAGAGTTCTAAGGATTGGTGGAA	GTCTACTTCCTCTGTGATGTTGT	XM_023647939.1	Exon 847/848 (R)	—	400/200

*Note:* F, forward primer; R, reverse primer. Primer concentrations are presented as forward/reverse (nM).

##### 2.6.3.3. Quantitative Real‐Time RT‐PCR Assay

Relative mRNA expression of selected genes was quantified using SYBR Green real‐time RT‐PCR on a LightCycler® 96 system (Roche, Germany). Each reaction contained RealQ Plus 2 × Master Mix Green, optimized concentrations of forward and reverse primers, diluted cDNA, and nuclease‐free water. The cycling protocol consisted of initial denaturation at 95°C for 15 min followed by 40 amplification cycles and melting‐curve analysis to confirm amplicon specificity. Relative expression ratios were calculated using amplification efficiency‐corrected Ct values normalized to the reference gene. Amplification efficiencies between 90% and 110% were considered acceptable [22].

### 2.7. Field Fertility Trial

For the field fertility trial, semen processed under the optimized freezing conditions was used for AI. A total of 16 broodmares, aged 5–14 years (mean ± SD: 8.1 ± 3.1), were inseminated using frozen semen obtained from two stallions. This trial was designed as a preliminary proof‐of‐concept to evaluate the practical applicability of the optimized cryopreservation protocol rather than to provide a definitive assessment of fertility outcomes. Therefore, the results should be interpreted with caution due to the limited number of stallions included. During the estrus period, all mares were subjected to regular rectal ultrasonographic assessments using a CTS990‐V SIUI rectal linear probe (7.5 MHz) until follicles with a mean diameter ≥ 37 mm and uterine edema ≥ Grade 2 were identified. At this stage, a single intravenous injection of 5000‐IU human chorionic gonadotropin (hCG; Karma, BSV BioScience GmbH) was administered. Ovulation monitoring commenced 24 h later, and mares were inseminated deep intracornually within 1‐2 h postovulation using a Minitube universal deep intrauterine insemination catheter. The insemination dose was standardized to 500 million total spermatozoa (equivalent to five semen straws) per mare. Following insemination, mares were re‐examined within 6–12 h to detect intrauterine fluid accumulation and treated as required. Pregnancy diagnosis was performed on Days 21 and 60 postinseminations.

### 2.8. Statistical Analysis

All statistical analyses were performed using IBM SPSS Statistics Version 26 (IBM Corp., Armonk, NY, USA), and graphs were prepared using GraphPad Prism Version 9 (GraphPad Software, USA). Data were assessed for normality using the Shapiro–Wilk test and for homogeneity of variances using Levene’s test. Variables that deviated from normality were log‐transformed when appropriate; where assumptions were not fully met, results were interpreted with caution. For all outcome variables, including sperm kinematic, functional, flow‐cytometric, oxidative stress, and gene expression parameters, linear mixed‐effects models were used to account for the hierarchical structure of the data. Treatment (osmolarity or cooling rate) was included as a fixed effect. For semen quality, flow‐cytometric, and oxidative stress variables, stallion and ejaculate were included as random effects to account for between‐animal and within‐animal variability. For gene expression data, analyses were performed separately for each gene, with treatment included as a fixed effect and stallion included as a random effect. Bonferroni‐adjusted pairwise comparisons were used for post hoc testing. Results are presented as mean ± standard error of the mean (SEM). Statistical significance was set at *p* ≤ 0.05 (two‐sided), and *p* values are reported together with the corresponding test statistics and estimated marginal means in accordance with SAMPL guidelines.

## 3. Results

### 3.1. Phase A: Effects of the Freezing Diluent Effective Osmotic Pressure on Frozen–Thawed Spermatozoa Characteristics

#### 3.1.1. Non‐Flowcytometric Assessments

##### 3.1.1.1. Osmolarity Measurements and Effective Tonicity

Measured osmolarity values were slightly lower than calculated targets across all freezing extenders (321.3 ± 3.2, 384.3 ± 4.0, and 440.0 ± 5.0 mOsm/kg for the 330, 390, and 450 groups, respectively). Following the two‐step (1:1) dilution, the final effective osmolarities decreased to 318.3 ± 3.5, 356.6 ± 7.6, and 383.0 ± 2.6 mOsm/kg, closely matching calculated values. Although total osmolarity markedly increased after glycerol addition (> 2000 mOsm/kg), these values do not represent effective tonicity due to the permeable nature of glycerol. Therefore, spermatozoa were effectively exposed to iso‐osmotic (∼318 mOsm/kg), moderately hypertonic (∼357 mOsm/kg), and highly hypertonic (∼383 mOsm/kg) conditions prior to freezing. These results confirm that the experimental design achieved distinct and controlled osmotic environments for subsequent analyses (Table 3).

**TABLE 3:** Calculated versus measured osmolarity of freezing extenders and final effective osmolarity after two‐step dilution.

Freezing extender group (mOsm/kg)	Sucrose (gr/50 mL)	Calculated osmolarity (mOsm/kg)	Measured osmolarity (mOsm/kg)	Calculated final effective osmolarity (mOsm/kg)	Measured final effective osmolarity (mOsm/kg)
330	0.87	330	321.3 ± 3.2	330	318.3 ± 3.5 (2011.3)
390	1.725	390	384.3 ± 4.0	360	356.6 ± 7.6 (2159.3)
450	2.585	450	440.0 ± 5.0	390	383.0 ± 2.6 (2205.3)

*Note:* Values are presented as mean ± SD (three independent measurements). Calculated osmolarity values represent target formulation levels based on extender composition. Measured osmolarity values were determined prior to glycerol addition. Measured final effective osmolarity represents the osmotic conditions experienced by spermatozoa after 1:1 dilution. Values in parentheses indicate total osmolarity after glycerol addition and reflect the contribution of permeable cryoprotectants; these do not represent effective tonicity.

##### 3.1.1.2. Sperm Kinematic Parameters

Osmolarity did not significantly affect any of the evaluated sperm kinematic parameters, including TM (F_2,37_ = 3.080, *p* = 0.058), PM (F_2,37_ = 2.464, *p* = 0.099), VCL (F_2,36.6_ = 2.458, *p* = 0.100), VAP (F_2,36_ = 2.457, *p* = 0.100), VSL (F_2,37.0_ = 3.070, *p* = 0.058), STR (F_2,37_ = 0.969, *p* = 0.389), LIN (F_2,37_ = 1.424, *p* = 0.254), or WOB (F_2,37_ = 0.171, *p* = 0.844). Similarly, sampling order (first versus second ejaculate) had no significant effect on any of these parameters (*p* > 0.05). Although not statistically significant, the highest osmolarity group showed a numerical decline in motility and velocity parameters compared with the 330 and 390 mOsm/kg groups. No significant pairwise differences were detected among osmolarity groups for any parameter (*p* > 0.05; Table 4).

**TABLE 4:** Effect of freezing extender osmolarity on post‐thaw sperm motility parameters.

Parameter	330 mOsm/kg	390 mOsm/kg	450 mOsm/kg
TM (%)	53.52 ± 3.45	53.01 ± 3.48	42.54 ± 3.44
PM (%)	23.23 ± 1.63	21.97 ± 1.58	18.17 ± 1.65
VCL (μm/s)	20.09 ± 1.93	18.09 ± 1.57	14.76 ± 1.54
VAP (μm/s)	10.95 ± 0.97	10.15 ± 0.86	8.18 ± 0.81
VSL (μm/s)	7.13 ± 0.61	6.72 ± 0.51	5.29 ± 0.48
STR (%)	65.33 ± 0.52	67.93 ± 2.63	65.24 ± 0.65
LIN (%)	36.18 ± 0.75	37.63 ± 0.91	36.53 ± 0.86
WOB (%)	55.30 ± 0.80	56.06 ± 1.40	55.89 ± 0.84

*Note:* Values are presented as mean ± SEM. Different superscripts (if present) within the same row indicate statistically significant differences between groups (*p* ≤ 0.05).

Abbreviations: LIN, linearity; PM, progressive motility; STR, straightness; TM, total motility; VAP, average path velocity; VCL, curvilinear velocity; VSL, straight‐line velocity; WOB, wobble.

##### 3.1.1.3. Functional and Structural Parameters

Osmolarity did not significantly affect general sperm parameters, including viability (F_2,38_ = 0.221, *p* = 0.803), membrane functional integrity (HOST; F_2,38_ = 0.043, *p* = 0.958), or morphology (F_2,38_ = 0.407, *p* = 0.668), and sampling order had no effect on these variables (*p* > 0.05). In contrast, osmolarity significantly influenced acrosome status. The proportion of noncapacitated spermatozoa (CTC‐F) was significantly higher at 390 mOsmol/kg compared with both 330 and 450 mOsmol/kg (*p* < 0.05), while the percentage of acrosome‐reacted spermatozoa (CTC‐AR) was significantly lower at 390 mOsmol/kg compared with the other groups (*p* < 0.05). No significant differences were observed in the proportion of capacitated spermatozoa (CTC‐B) among groups (F_2,36_ = 0.209, *p* = 0.813). Similarly, sperm DNA integrity was significantly affected by osmolarity. The percentage of spermatozoa with intact DNA (DNAL) was significantly higher at both 390 and 450 mOsmol/kg compared with 330 mOsmol/kg (*p* < 0.001), whereas no difference was observed between 390 and 450 mOsmol/kg (*p* > 0.05). Conversely, the proportions of spermatozoa with fragmented (DNAS) and degraded DNA (DNAN) were significantly higher at 330 mOsmol/kg compared with the hypertonic groups (*p* < 0.05), with no differences between 390 and 450 mOsmol/kg. Overall, these findings indicate that while increasing extender osmolarity did not affect basic sperm structural parameters, a moderately hypertonic environment (∼390 mOsmol/kg) improved acrosome integrity and reduced DNA damage, whereas iso‐osmotic conditions were associated with higher levels of acrosome reaction and DNA fragmentation (Table 5).

**TABLE 5:** Effect of freezing extender osmolarity on post‐thaw sperm functional and structural characteristics.

Parameter	330 mOsm/kg	390 mOsm/kg	450 mOsm/kg
*General sperm parameters*			
Viability (%)	53.65 ± 3.05^a^	51.65 ± 2.98^a^	52.50 ± 2.26^a^
HOST (%)	29.13 ± 2.59^a^	29.49 ± 3.38^a^	29.73 ± 4.00^a^
Morphology (%)	94.65 ± 0.53^a^	95.15 ± 0.53^a^	94.90 ± 0.34^a^

*Acrosome status (CTC assay)*			
Noncapacitated (CTC‐F, %)	52.32 ± 1.69^a^	64.05 ± 2.54^b^	57.75 ± 2.11^a^
Capacitated (CTC‐B, %)	2.10 ± 0.43^a^	2.33 ± 0.45^a^	2.25 ± 0.51^a^
Acrosome‐reacted (CTC‐AR, %)	45.58 ± 1.71^a^	33.72 ± 2.66^b^	40.00 ± 1.95^a^

*DNA integrity (SCD assay)*			
Intact DNA (Large halo, %)	64.38 ± 2.37^a^	75.58 ± 1.77^b^	79.81 ± 1.30^b^
Fragmented DNA (Small halo, %)	19.99 ± 1.28^b^	13.67 ± 0.71^a^	11.00 ± 0.49^a^
Degraded DNA (No halo, %)	15.63 ± 1.61^b^	10.75 ± 1.46^a^	9.19 ± 1.08^a^

*Note:* Values are presented as mean ± SEM (*n* = 3). Different superscripts within the same row indicate statistically significant differences between groups (*p* ≤ 0.05).

Abbreviations: CTC, chlortetracycline assay (F, noncapacitated; B, capacitated; AR, acrosome‐reacted); HOST, hypo‐osmotic swelling test; SCDA, sperm chromatin dispersion assay.

##### 3.1.1.4. MDA: Oxidative Stress Marker

Osmolarity did not significantly affect MDA levels (F_2,26_ = 1.740, *p* = 0.195), and sampling order showed only a nonsignificant trend (*p* = 0.058). No significant pairwise differences were detected among osmolarity groups (*p* > 0.05).

#### 3.1.2. Flowcytometric Assessments

##### 3.1.2.1. Annexin V/Propidium Iodide Apoptosis Assay

Extender osmolarity significantly affected sperm apoptotic status in a pattern‐dependent manner, whereas sampling (first vs. second ejaculate) had no significant effect on most parameters and no significant interaction with osmolarity was detected (*p* > 0.05). The proportion of live, nonapoptotic spermatozoa (An−/PI−) was significantly affected by osmolarity (F_2,16_ = 23.837, *p* < 0.001). Pairwise comparisons showed that values were significantly higher at 390 mOsm/kg than at 330 mOsm/kg (*p* < 0.001) and numerically higher than at 450 mOsm/kg; however, the difference between 390 and 450 mOsm/kg was not statistically significant (*p* > 0.05). No significant difference was observed between 330 and 450 mOsm/kg (*p* > 0.05). However, sampling had no significant effect on this parameter (F_1,4_ = 0.574, *p* = 0.491). Early apoptotic sperm (An+/PI−) were significantly affected by osmolarity (F_2,16.000_ = 19.430, *p* < 0.001), with lower values at 450 mOsm/kg compared with both 330 (*p* = 0.001) and 390 mOsm/kg (*p* < 0.001), while no difference was observed between 330 and 390 mOsm/kg (*p* > 0.05); sampling did not influence this parameter (F_1,8_ = 0.741, *p* = 0.414). In contrast, late apoptotic sperm (An+/PI+) were significantly influenced by osmolarity (F_2,20.000_ = 5.916, *p* = 0.010), with higher values at 330 mOsm/kg compared with 390 mOsm/kg (*p* = 0.011), while no differences were observed between other groups (*p* > 0.05); sampling had no effect (F_1,20_ = 0.000, *p* = 0.983). The proportion of early necrotic sperm (An−/PI+) was significantly affected by both sampling (F_1,24_ = 4.618, *p* = 0.042) and osmolarity (F_2,24_ = 12.573, *p* < 0.001), with higher values in the second ejaculate compared with the first and higher values at 450 mOsm/kg compared with 390 mOsm/kg (*p* < 0.001), while no interaction effect was detected (*p* > 0.05). Similarly, total dead sperm (PI+) were significantly influenced by osmolarity (F_2,16_ = 9.430, *p* = 0.002), with lower values at 390 mOsm/kg compared with both 330 (*p* = 0.016) and 450 mOsm/kg (*p* = 0.002), whereas sampling had no significant effect (F_1,8_ = 1.300, *p* = 0.287). These findings indicate that osmolarity, particularly 390 mOsm/kg, optimizes sperm viability and reduces apoptosis and membrane damage, while sampling effects are limited to necrotic spermatozoa (Table 6 and Supporting Figure 1).

**TABLE 6:** Effect of freezing extender osmolarity on sperm apoptosis and necrosis patterns.

Parameter	330 mOsm/kg	390 mOsm/kg	450 mOsm/kg
Live/nonapoptotic (An−/PI−, %)	42.17 ± 4.92^a^	57.93 ± 3.88^b^	43.80 ± 1.47^a^
Early apoptotic (An+/PI−, %)	9.55 ± 2.14^ab^	10.83 ± 1.48^a^	4.25 ± 1.37^b^
Late apoptotic (An+/PI+, %)	29.90 ± 0.61^a^	23.77 ± 3.15^b^	28.50 ± 3.30^ab^
Necrotic (An−/PI+, %)	18.38 ± 6.38^ab^	7.47 ± 1.68^a^	23.45 ± 3.85^b^
Total dead (PI+, %)	48.28 ± 6.73^a^	36.65 ± 6.39^b^	51.49 ± 0.70^a^

*Note:* Values are presented as mean ± SEM (*n* = 3). Different superscripts within the same row indicate statistically significant differences between osmolarity groups (*p* ≤ 0.05; linear mixed model with Bonferroni‐adjusted pairwise comparisons). Sampling (first vs. second ejaculate) had no significant effect on most parameters (*p* > 0.05), except for necrotic sperm (An−/PI+), where a significant sampling effect was observed (*p* = 0.042). No significant interaction between sampling and osmolarity was detected (*p* > 0.05).

Abbreviations: An, annexin V; PI, propidium iodide.

##### 3.1.2.2. Rhodamine 123 Staining Assay

Osmolarity significantly affected mitochondrial activity of spermatozoa. The proportion of mitochondria‐active sperm (Rh+/PI−) was significantly influenced by osmolarity (F_2,20_ = 33.870, *p* < 0.001), with higher values at 390 mOsm/kg compared with 330 (*p* = 0.031) and 450 mOsm/kg (*p* < 0.001) and higher values at 330 compared with 450 mOsm/kg (*p* < 0.001). However, mitochondrial activity was not affected by sampling or the interaction between factors (*p* > 0.05). In contrast, the proportion of mitochondria‐inactive viable sperm (Rh−/PI−) was not significantly influenced by osmolarity, sampling, or their interaction (F_2,24_ = 0.827, *p* = 0.450; *p* > 0.05 for all comparisons). These results suggest that mitochondrial function is primarily modulated by osmotic conditions, with optimal activity observed at 390 mOsm/kg (Table 7 and Supporting Figure 2).

**TABLE 7:** Effect of freezing extender osmolarity on sperm mitochondrial activity (rhodamine 123/PI staining).

Parameter	330 mOsm/kg	390 mOsm/kg	450 mOsm/kg
Functional mitochondria (Rh+/PI−, %)	47.97 ± 5.17^b^	58.80 ± 7.03^a^	27.70 ± 2.29^c^
Nonfunctional mitochondria (Rh−/PI−, %)	9.30 ± 1.23^a^	9.71 ± 1.69^a^	13.54 ± 7.54^a^

*Note:* Values are presented as mean ± SEM (*n* = 3). Different superscripts within the same row indicate statistically significant differences between osmolarity groups (*p* ≤ 0.05; linear mixed model with Bonferroni‐adjusted pairwise comparisons). Sampling (first vs. second ejaculate) had no significant effect on mitochondrial parameters (*p* > 0.05), and no significant interaction was detected (*p* > 0.05).

Abbreviations: PI, propidium iodide; Rh, rhodamine 123.

##### 3.1.2.3. FITC‐PNA Staining Assay

Extender osmolarity significantly influenced acrosome integrity, consistent with the significant osmolarity × pattern interaction (*F*_16,243_ = 12.135, *p* < 0.001). The proportion of spermatozoa with intact acrosomes (PNA–/PI–) was significantly lower at 450 mOsm/kg compared with both 330 and 390 mOsm/kg (*p* < 0.001), whereas no difference was observed between 330 and 390 mOsm/kg (*P* > 0.05). In contrast, the proportion of spermatozoa with reacted acrosomes (PNA+/PI–) was significantly higher at 450 mOsm/kg compared with both 330 and 390 mOsm/kg (*p* < 0.001), with no difference between the latter two groups (*p* > 0.05). These results indicate that elevated osmolarity compromises acrosomal stability and promotes premature acrosome reaction (Table 8 and Supporting Figure 3).

**TABLE 8:** Effect of freezing extender osmolarity on sperm acrosome integrity (FITC‐PNA/PI staining).

Parameter	330 mOsm/kg	390 mOsm/kg	450 mOsm/kg
Intact acrosome (PNA−/PI−, %)	44.10 ± 3.18^a^	51.68 ± 6.11^a^	26.72 ± 2.91^b^
Reacted acrosome (PNA+/PI−, %)	30.27 ± 5.94^a^	16.40 ± 5.88^a^	49.00 ± 3.87^b^

*Note:* Values are presented as mean ± SEM (*n* = 3). Different superscripts within the same row indicate statistically significant differences between osmolarity groups (*p* ≤ 0.05; linear mixed model with Bonferroni‐adjusted pairwise comparisons). Sampling (first vs. second ejaculate) had no significant effect on acrosome integrity parameters (*p* > 0.05), and no significant interaction was detected (*p* > 0.05).

Abbreviations: PI, propidium iodide; PNA, peanut agglutinin.

#### 3.1.3. Transcriptomic Assays

##### 3.1.3.1. PCR Primers, Amplification Optimization, and Efficiency Calculation

Table 2 summarizes the information of the primer characteristics and concentration optimization results. Based on our result, all amplification efficiencies were between 90 and 110 percent. The melting curve analysis of the PCR products produced the specific single peaks according to the melting point of the PCR products.

##### 3.1.3.2. Relative Abundance of the Fertility‐Related Transcriptomic Markers

**FIGURE 3:**
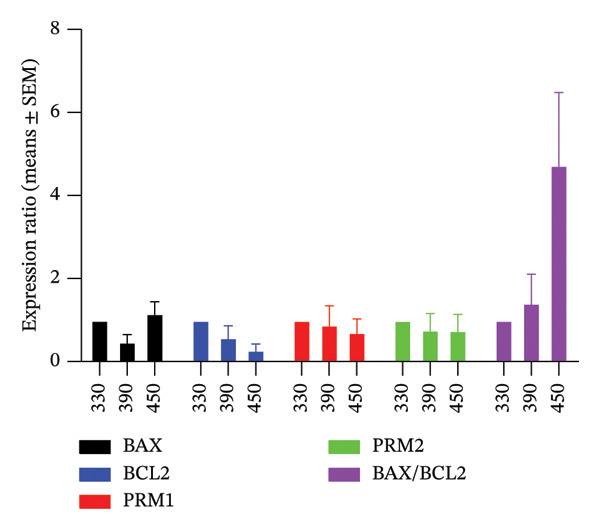
Effect of extender osmolarity (330, 390, and 450 mOsm/kg) on the relative expression of BAX, BCL2, PRM1, PRM2, and the BAX/BCL2 ratio in stallion sperm. Gene expression is presented as fold change relative to the 330 mOsm/kg group. Data are shown as estimated marginal means ± SEM. No significant differences were detected among osmolarity groups (*p* > 0.05).

Linear mixed model analysis revealed no significant effect of osmolarity on the expression of BAX (F_2,8_ = 2.13, *p* = 0.181), BCL2 (F_2,8_ = 2.42, *p* = 0.151), PRM1 (F_2,12_ = 0.21, *p* = 0.812), or PRM2 (F_2,8_ = 0.28, *p* = 0.765). Similarly, the BAX/BCL2 expression ratio was not significantly affected by osmolarity (F_2,12_ = 1.03, *p* = 0.385). Although not statistically significant, the BAX/BCL2 ratio showed a progressive numerical increase at higher osmolarities, suggesting a possible shift toward a proapoptotic profile under higher osmotic conditions; however, substantial interindividual variability limited statistical significance (Figure 3).

### 3.2. Part B: Effects of the Prefreezing Cooling Rates on Frozen–Thawed Spermatozoa Characteristics

#### 3.2.1. Non‐Flowcytometric Assessments

##### 3.2.1.1. Sperm Kinematic Parameters

Prefreezing cooling time significantly affected several post‐thaw sperm kinematic parameters. TM was significantly lower after 45 min of cooling than after 1.5 h and 3 h (F_2,18_ = 4.708, *p* = 0.023), with estimated marginal means of 42.85 ± 4.26, 59.38 ± 4.26, and 58.30 ± 4.26, respectively. PM was also significantly influenced by cooling time (F_2,18_ = 4.491, *p* = 0.026), being lower at 45 min (19.56 ± 3.45) than at 1.5 h (28.52 ± 3.45) and 3 h (27.29 ± 3.45). Similarly, VCL and VSL were significantly reduced in the 45‐min group compared with the longer cooling periods (VCL: F_2,18_ = 3.927, *p* = 0.038; VSL: F_2,18_ = 4.657, *p* = 0.023). In contrast, VAP showed only a numerical increase with prolonged cooling but did not reach statistical significance (F_2,18_ = 3.348, *p* = 0.058). Among trajectory parameters, WOB was significantly affected by cooling time (F_2,18_ = 5.080, *p* = 0.018), whereas STR and LIN were not significantly altered (*p* > 0.05). Sampling order had no significant effect on any kinematic variable (*p* > 0.05). Overall, these findings indicate that extending the prefreezing cooling period from 45 min to 1.5–3 h improved post‐thaw sperm motility and velocity characteristics, while no further significant improvement was observed between 1.5 h and 3 h (Table 9).

**TABLE 9:** Effects of prefreezing cooling rates on frozen–thawed spermatozoa motility parameters.

Parameter	45 min	1.5 h	3 h
TM (%)	45.50 ± 2.68^a^	52.30 ± 2.47^b^	54.90 ± 2.61^b^
PM (%)	18.90 ± 1.21^a^	23.70 ± 1.38^b^	24.80 ± 1.52^b^
VCL (μm/s)	16.70 ± 1.32^a^	19.80 ± 1.44^b^	20.60 ± 1.36^b^
VAP (μm/s)	8.90 ± 0.74^a^	10.80 ± 0.82^a^	11.20 ± 0.79^a^
VSL (μm/s)	5.10 ± 0.42^a^	6.80 ± 0.53^b^	7.00 ± 0.49^b^
STR (%)	65.10 ± 1.02^a^	66.40 ± 1.15^a^	67.20 ± 1.09^a^
LIN (%)	34.80 ± 0.88^a^	36.90 ± 0.91^a^	37.10 ± 0.95^a^
WOB (%)	56.80 ± 1.12^a^	55.20 ± 1.08^ab^	53.90 ± 1.05^b^

*Note:* Different letters in superscripts within rows indicate significant differences (*p* ≤ 0.05). Data are presented as mean ± SEM.

Abbreviations: LIN, linearity; PM, progressive motility; STR, straightness; TM, total motility; VAP, average path velocity; VCL, curvilinear velocity; VSL, straight‐line velocity; WOB, wobble.

##### 3.2.1.2. Functional and Structural Parameters

Prefreezing cooling time significantly affected some general sperm parameters, while others remained unchanged. Sperm viability was significantly influenced by cooling time (F_2,18_ = 7.539, *p* = 0.004), with lower values at 45 min compared with 1.5 h and 3 h, whereas no difference was observed between the two longer cooling durations. In contrast, membrane functional integrity (HOST; F_2,18_ = 1.985, *p* = 0.166) and morphology (*p* > 0.05) were not significantly affected, and the sampling order had no effect on these variables (*p* > 0.05). Cooling time also significantly influenced acrosome status. The proportion of CTC‐F (F_2,18_ = 37.021, *p* < 0.001) was significantly higher at 1.5 h and 3 h compared with 45 min, while the percentage of CTC‐AR (F_2,18_ = 33.522, *p* < 0.001) was significantly lower at longer cooling durations. No significant differences were observed in the proportion of CTC‐B (F_2,18_ = 1.491, *p* = 0.252). Similarly, sperm DNA integrity was significantly affected by cooling time. The percentage of spermatozoa with intact DNA (DNAL; F_2,18_ = 3.962, *p* = 0.038) was significantly higher at 3 h compared with 45 min, whereas no difference was observed between the two longer cooling periods (*p* > 0.05). Conversely, the proportion of spermatozoa with fragmented DNA (DNAS; F_2,18_ = 5.294, *p* = 0.016) was significantly higher at 45 min compared with the longest cooling duration, while degraded DNA (DNAN; F_2,18_ = 0.090, *p* = 0.915) did not differ among groups. Overall, these findings indicate that extending the cooling period improved sperm viability, acrosome stability, and DNA integrity, whereas shorter cooling (45 min) was associated with increased acrosome reaction and DNA fragmentation (Table 10).

**TABLE 10:** Effect of the prefreezing cooling rate on post‐thaw sperm functional and structural characteristics.

Parameter	45 min	1.5 h	3 h
*General sperm parameters*			
Viability (%)	47.80 ± 1.65^a^	58.10 ± 2.69^b^	60.00 ± 2.66^b^
HOST (%)	35.30 ± 2.60^a^	39.90 ± 2.98^a^	42.40 ± 3.29^a^
Morphology (%)	93.70 ± 0.70^a^	95.40 ± 0.72^a^	94.20 ± 0.77^a^

*Acrosome status (CTC assay)*			
Noncapacitated (CTC‐F, %)	48.70 ± 1.39^a^	60.90 ± 2.27^b^	66.10 ± 1.18^c^
Capacitated (CTC‐B, %)	4.00 ± 0.37^a^	4.00 ± 0.42^a^	3.10 ± 0.38^a^
Acrosome‐reacted (CTC‐AR, %)	47.30 ± 1.51^a^	35.10 ± 2.13^b^	30.80 ± 1.26^b^

*DNA integrity (SCD assay)*			
Intact DNA (Large halo, %)	69.60 ± 0.99^a^	73.30 ± 1.96^ab^	75.80 ± 1.43^b^
Fragmented DNA (Small halo, %)	17.90 ± 1.60^a^	14.80 ± 0.83^ab^	12.90 ± 0.57^b^
Degraded DNA (No halo, %)	12.50 ± 1.79^a^	11.90 ± 1.67^a^	11.30 ± 1.83^a^

*Note:* Values are presented as mean ± SEM. Different superscripts within the same row indicate statistically significant differences between groups (*p* ≤ 0.05).

Abbreviations: CTC, chlortetracycline assay (F, noncapacitated; B, capacitated; AR, acrosome‐reacted); HOST, hypo‐osmotic swelling test; SCDA, sperm chromatin dispersion assay.

##### 3.2.1.3. MDA: Oxidative Stress Marker

MDA levels showed a numerical decrease as cooling duration increased from 45 min to 3 h; however, this difference was not statistically significant (*p* > 0.05).

#### 3.2.2. Flowcytometric Assessments

##### 3.2.2.1. Annexin V/Propidium Iodide Apoptosis Assay

Prefreezing cooling rate significantly affected sperm apoptotic status in a pattern‐dependent manner. The proportion of live, nonapoptotic spermatozoa (An−/PI−) was significantly influenced by the cooling rate (*F*_2,22.764_ = 11.869,  *p* < 0.001), with lower values at 45 min compared with both 1.5 h (*p* = 0.009) and 3 h (*p* < 0.001), while no difference was observed between 1.5 h and 3 h (*p* = 0.492). Early apoptotic sperm (An+/PI−) were not affected by the cooling rate (*F*_2,24.000_ = 2.775,  *p* = 0.082), with no differences among groups (*p* > 0.05). In contrast, late apoptotic sperm (An+/PI+) were significantly higher at 45 min compared with both 1.5 h and 3 h (*F*_2,19.067_ = 26.624, *p* < 0.001; *p* < 0.001 for both comparisons), with no difference between the latter two (*p* = 0.158). The proportion of early necrotic sperm (An−/PI+) was not affected by the cooling rate (*F*_2,19.908_ = 0.836,  *p* = 0.448). However, total dead sperm (PI+) were significantly higher at 45 min compared with 3 h (*F*_2,18.947_ = 5.188,  *p* = 0.016;  *p* = 0.015), with no differences between other groups (*p* > 0.05). These findings indicate that longer cooling durations reduce apoptosis progression and overall cell death (Table 11).

**TABLE 11:** Frozen–thawed spermatozoa apoptosis/necrosis patterns in different cooling rate groups.

Parameter	45 min	1.5 h	3 h
Live/nonapoptotic (An−/PI−, %)	39.57 ± 3.65^a^	48.59 ± 1.05^b^	52.52 ± 1.49^b^
Early apoptotic (An+/PI−, %)	10.95 ± 0.93^a^	6.75 ± 1.78^a^	6.81 ± 1.19^a^
Late apoptotic (An+/PI+, %)	31.01 ± 0.83^a^	22.83 ± 0.92^b^	19.48 ± 1.55^b^
Necrotic (An−/PI+, %)	18.47 ± 2.30^a^	21.82 ± 2.04^a^	21.19 ± 1.07^a^
Total dead (PI+, %)	49.49 ± 2.91^a^	43.75 ± 1.58^ab^	40.49 ± 1.06^b^

*Note:* Values are presented as mean ± SEM. Different superscripts within the same row indicate statistically significant differences between groups (*p* ≤ 0.05; linear mixed model with Bonferroni‐adjusted pairwise comparisons).

Abbreviations: An, annexin V; PI, propidium iodide.

##### 3.2.2.2. Rhodamine 123 Staining Assay

Prefreezing cooling rate significantly affected the sperm mitochondrial activity. The proportion of spermatozoa with functional mitochondria (Rh+/PI−) was significantly lower at 45 min compared with both 1.5 h and 3 h (*F*_2,31.447_ = 21.265,  *p* < 0.001;  *p* < 0.001 for both comparisons), while no difference was observed between 1.5 h and 3 h (*p* = 0.347). Conversely, the proportion of spermatozoa with nonfunctional mitochondria (Rh−/PI−) was significantly higher at 45 min compared with both 1.5 h (*p* = 0.009) and 3 h (*p* = 0.001)(*F*_2,19.967_ = 10.781,  *p* = 0.001), with no difference between the latter groups (*p* = 0.936). These results demonstrate that longer cooling durations improve mitochondrial integrity (Table 12).

**TABLE 12:** Effect of prefreezing cooling rate on sperm mitochondrial activity (rhodamine 123/PI staining).

Parameter	45 min	1.5 h	3 h
Functional mitochondria (Rh+/PI−, %)	29.70 ± 5.50^a^	53.33 ± 2.35^b^	61.53 ± 3.69^b^
Nonfunctional mitochondria (Rh−/PI−, %)	16.40 ± 2.88^a^	8.14 ± 0.74^b^	5.60 ± 0.52^b^

*Note:* Values are presented as mean ± SEM. Different superscripts within the same row indicate statistically significant differences between groups (*p* ≤ 0.05; linear mixed model with Bonferroni‐adjusted pairwise comparisons).

Abbreviations: PI, propidium iodide; Rh, rhodamine 123.

##### 3.2.2.3. FITC‐PNA Staining Assay

Prefreezing cooling rate significantly influenced sperm acrosome integrity. The proportion of spermatozoa with reacted acrosomes (PNA+/PI−) was significantly higher at 45 min compared with both 1.5 h (*p* = 0.003) and 3 h (*P* = 0.032)(*F*_2,18_ = 8.370,  *p* = 0.003), whereas no difference was observed between 1.5 h and 3 h (*p* = 0.837). In contrast, the proportion of spermatozoa with intact acrosomes (PNA−/PI−) was significantly lower at 45 min compared with 3 h (*p* = 0.046)(*F*_2,18_ = 4.207,  *p* = 0.032), with no differences between 45 min and 1.5 h or between 1.5 h and 3 h (*p* > 0.05). These findings indicate that longer cooling durations enhance acrosomal stability and reduce premature acrosome reaction (Table 13).

**TABLE 13:** Effect of prefreezing cooling rate on sperm acrosome integrity (FITC‐PNA/PI staining).

Parameter	45 min	1.5 h	3 h
Intact acrosome (PNA−/PI−, %)	32.68 ± 1.23^a^	46.68 ± 5.75^ab^	48.90 ± 3.93^b^
Acrosome‐reacted (PNA+/PI−, %)	44.99 ± 4.52^a^	18.90 ± 4.68^b^	26.25 ± 5.87^b^

*Note:* Values are presented as mean ± SEM. Different superscripts within the same row indicate statistically significant differences between groups (*p* ≤ 0.05; linear mixed model with Bonferroni‐adjusted pairwise comparisons).

Abbreviations: PI, propidium iodide; PNA, peanut agglutinin.

#### 3.2.3. Transcriptomic Assays

##### 3.2.3.1. Relative Abundance of the Fertility Related Transcriptomic Markers

**FIGURE 4:**
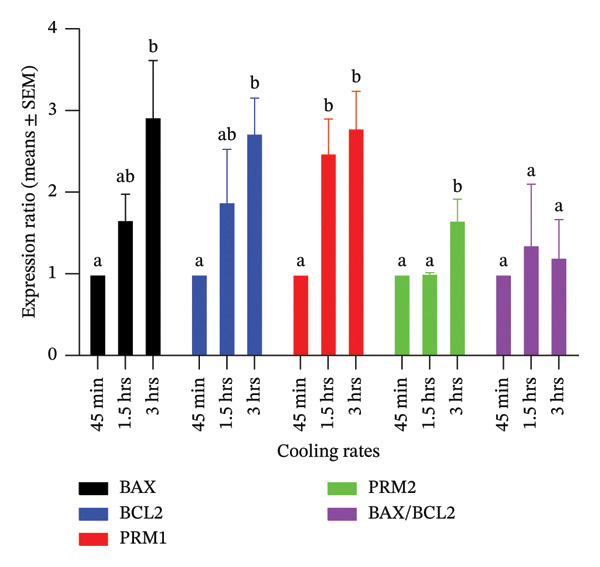
Effect of cooling rate (45 min, 1.5 h, and 3 h) on the relative expression of BAX, BCL2, PRM1, PRM2, and the BAX/BCL2 ratio in stallion sperm. Gene expression is presented as fold change (2^−ΔΔCt^) relative to the 45‐min group. Data are shown as estimated marginal means ± SEM. Different letters (a, b) indicate significant differences among cooling rate groups within each gene (*p* < 0.05).

Linear mixed‐model analysis performed separately for each gene revealed a significant effect of cooling rate on the expression of BAX (F_2,6_ = 8.13, *p* = 0.020), BCL2 (F_2,6_ = 7.11, *p* = 0.026), PRM1 (F_2,6_ = 15.08, *p* = 0.005), and PRM2 (F_2,9_ = 6.65, *p* = 0.017). Estimated marginal means demonstrated a progressive increase in expression with slower cooling rates, rising from 1.00 ± 0.43 to 2.93 ± 0.43 for BAX, 1.00 ± 0.44 to 2.73 ± 0.44 for BCL2, 1.00 ± 0.35 to 2.80 ± 0.35 for PRM1, and 1.00 ± 0.15 to 1.66 ± 0.15 for PRM2 between 45 min and 3 h. Bonferroni‐adjusted pairwise comparisons indicated that expression levels at 3 h were significantly higher than at 45 min for BAX (*p* = 0.022), BCL2 (*p* = 0.028), PRM1 (*p* = 0.006), and PRM2 (*p* = 0.033), whereas differences between 1.5 h and 3 h were not consistently significant (*p* > 0.05). In contrast, analysis of the BAX/BCL2 expression ratio showed no significant effect of cooling rate (F_2,6_ = 0.71, *p* = 0.529), with estimated marginal means of 1.00 ± 0.48, 1.60 ± 0.48, and 1.28 ± 0.48 for 45 min, 1.5 h, and 3 h, respectively, indicating that although both pro‐ and antiapoptotic genes were upregulated with slower cooling, their relative balance remained unchanged (Figure 4).

#### 3.2.4. Field Fertility Trial

Using semen cryopreserved under the optimized conditions, pregnancy was achieved in 12 out of 16 mares (75.0%) at Day 21 and in 10 out of 16 mares (62.5%) at Day 60 following the first insemination. The results of the fertility trial are presented in Table 14. Pregnancy rates represent per‐cycle outcomes following a single insemination.

**TABLE 14:** Pregnancy outcomes following artificial insemination with frozen–thawed semen.

Mare ID	Stallion	Age (years)	Pregnancy (Day 21)	Pregnancy (Day 60)
1	M	4	Negative	—
2	M	12	Positive	Positive
3	M	6	Positive	Positive
4	M	14	Negative	—
5	M	8	Positive	Positive
6	M	9	Positive	Negative
7	M	7	Positive	Positive
8	M	12	Negative	—
9	E	4	Positive	Positive
10	E	8	Positive	Positive
11	E	12	Negative	—
12	E	9	Positive	Positive
13	E	9	Positive	Negative
14	E	7	Positive	Positive
15	E	4	Positive	Positive
16	E	5	Positive	Positive

*Note:* Positive = pregnant; Negative = nonpregnant. M and E indicate stallion codes.

## 4. Discussion

The present study provides a comprehensive evaluation of two critical physicochemical determinants of stallion semen cryosurvival—freezing extender tonicity and prefreezing cooling rate—under a practical, field‐applicable framework. By explicitly controlling effective osmotic pressure (tonicity) and integrating it with a standardized manual cooling approach, this study addresses key limitations in previous research where osmolarity was often confounded by permeating cryoprotectants and cooling conditions were inconsistently applied. Our findings demonstrate that a moderately hypertonic environment (∼360 mOsmol/kg) combined with controlled slow cooling (1.5–3 h) optimizes post‐thaw sperm function across structural and functional endpoints, while excessive osmotic stress or rapid cooling induces measurable cellular damage.

The beneficial effects of moderate hypertonicity observed in this study are consistent with established cryobiological principles. Exposure to slightly hypertonic conditions prior to freezing promotes controlled cellular dehydration, reducing intracellular water content and thereby limiting intracellular ice formation during the freezing process. This mechanism likely contributed to the observed improvements in acrosome integrity, mitochondrial activity, and DNA stability. Similar protective effects of nonpermeating sugars, such as sucrose and trehalose, have been reported in stallion sperm and other species, where they stabilize membrane phospholipids and mitigate phase transitions during cooling [6–8]. Importantly, our study extends these findings by demonstrating that the protective effect is primarily dependent on effective tonicity rather than total osmolarity, highlighting the importance of distinguishing between permeating and nonpermeating solutes in cryopreservation systems. This distinction is particularly relevant when interpreting previous studies that have evaluated the addition of exogenous compounds to semen extenders.

An important consideration arising from the present study relates to the interpretation of previous reports evaluating the addition of nonpermeating sugars or other supplements in semen extenders. Numerous studies have reported beneficial effects of compounds such as sucrose and trehalose on sperm cryosurvival [6–8], as well as the use of antioxidants, amino acids, and metabolic substrates including L‐carnitine and pyruvate [23, 24]. However, in many of these studies, the final osmolarity or effective tonicity of the extender was not measured or reported, making it difficult to distinguish between solute‐specific effects and osmotic effects. Because nonpermeating solutes directly contribute to the effective osmotic pressure of the medium, their addition inevitably alters sperm cell volume regulation and dehydration dynamics prior to freezing [3, 9]. Consequently, at least part of the reported protective effects may be mediated by changes in tonicity rather than intrinsic biochemical properties of the added compounds. This limitation is further compounded by the use of different base extenders with variable initial osmolarities across studies, meaning that identical nominal concentrations of additives (e.g., 50‐mM sucrose or trehalose) may result in substantially different osmotic environments. A similar concern applies to studies evaluating antioxidants or amino acids, particularly when these compounds are present at concentrations sufficient to influence osmotic balance. The present study addresses this gap by explicitly controlling and reporting effective tonicity, thereby enabling a clearer distinction between osmotic and compositional effects. These findings highlight the importance of standardizing and reporting osmotic conditions in future cryopreservation studies to improve interpretability and reproducibility.

In contrast, excessive hypertonicity (∼390 mOsmol/kg effective exposure) resulted in detrimental effects, including increased necrosis and acrosomal damage, and a tendency toward an increased Bax/Bcl2 ratio. These findings suggest that excessive osmotic stress disrupts membrane integrity and may promote apoptotic processes, likely through osmotic imbalance–induced oxidative stress and mitochondrial dysfunction. The observed cellular damage is consistent with previous reports linking high osmotic pressure to increased reactive oxygen species (ROS) generation, lipid peroxidation, and membrane destabilization in spermatozoa [23, 25]. Thus, our results support the concept that the beneficial effects of osmotic manipulation are confined to a narrow physiological window, beyond which cellular damage outweighs cryoprotective benefits.

The effects of prefreezing cooling rate observed in this study further emphasize the importance of membrane biophysics during cryopreservation. Extending the cooling period from 45 min to 1.5–3 h significantly improved motility, viability, mitochondrial function, acrosome stability, and DNA integrity, while reducing apoptosis and total cell death. These improvements can be attributed to a more gradual transition of membrane lipids from the liquid‐crystalline to gel phase, allowing adequate time for membrane reorganization and stabilization. Rapid cooling, in contrast, likely induces cold shock, leading to membrane destabilization, impaired mitochondrial function, and increased susceptibility to oxidative damage [10, 11].

Notably, the absence of significant differences between 1.5 and 3‐h cooling durations suggests the existence of a plateau effect, beyond which further slowing of cooling confers no consistent additional benefit. This finding has important practical implications, indicating that extending cooling beyond a critical duration may not be necessary for achieving optimal cryosurvival. From a field perspective, this supports the use of moderate‐duration manual cooling protocols as an effective alternative to programmable freezing systems.

The integration of molecular findings should be interpreted with caution. Although functional improvements in DNA integrity were evident, the expression levels of BAX, BCL2, PRM1, and PRM2 were not significantly affected by treatments, and the observed increase in the Bax/Bcl2 ratio under higher osmotic conditions was not statistically significant. This suggests that post‐thaw functional changes are primarily driven by structural and physiological alterations rather than major shifts in gene expression. Given the transcriptionally inactive nature of mature spermatozoa, detected transcripts likely reflect residual mRNA rather than active gene regulation and, therefore, may not directly correspond to functional outcomes.

Although oxidative stress, as assessed by MDA levels, was not significantly affected by treatments, the numerical trends and concurrent apoptotic changes suggest that oxidative mechanisms may still contribute to cryoinjury but were not fully captured by this endpoint alone. Future studies incorporating additional oxidative stress markers or real‐time ROS measurements may provide further insight into these dynamics.

Importantly, the preliminary fertility trial demonstrated that semen processed under the optimized conditions achieved pregnancy rates of 75.0% at Day 21 and 62.5% at Day 60, which fall within or exceed the range commonly reported for frozen–thawed stallion semen under field conditions. While these results should be interpreted cautiously due to the limited number of stallions and mares, they provide valuable proof‐of‐concept evidence that improvements in post‐thaw sperm quality can translate into acceptable reproductive performance.

Despite these strengths, several limitations should be acknowledged. Although stallion and ejaculate variability were partially accounted for in the experimental design, their effects were not fully modeled in all analyses and may have influenced some outcomes. The fertility trial was limited in scale and included semen from only two stallions, restricting the generalizability of the results. Additionally, potential interactions between osmotic conditions and cooling rates were not evaluated and warrant further investigation. Finally, the study did not directly assess membrane phase behavior or intracellular ice formation, which could provide further mechanistic insight into the observed effects.

In conclusion, this study demonstrates that precise control of effective tonicity, combined with controlled prefreezing cooling, is critical for optimizing stallion sperm cryosurvival. A moderately hypertonic environment (∼360 mOsmol/kg) together with a practical slow cooling protocol (1.5–3 h) enhances sperm structural integrity and functional competence, ultimately supporting acceptable fertility outcomes. These findings provide a scientifically grounded and field‐applicable framework for improving stallion semen cryopreservation and highlight the importance of integrating physicochemical and biological factors in the design of cryopreservation protocols.

## Author Contributions

Leili Mavalizadeh carried out the research project, analyzed the results, and prepared the manuscript. Mohammad Reza Divar proposed the research idea, carried out the research project, analyzed the results, and prepared the manuscript. Asghar Mogheiseh carried out the research project, analyzed the results, and prepared the manuscript. Maryam Emami carried out the research project.

## Funding

This work was supported financially by Shiraz University, Veterinary School, Andrology Laboratory under grant number 0GCB1M369962, 2020.

## Ethics Statement

The current study received ethical approval from the Iranian laboratory animal ethics framework, under the oversight of the Iranian Society for the Prevention of Cruelty to Animals and the Shiraz University Research Council (IACUC no: 4687/63). As the corresponding author, I hereby affirm, on behalf of all researchers engaged in this study, that we have collectively granted our informed consent to participate. Additionally, before the commencement of data collection, a comprehensive overview of the study’s aims, methodologies, potential risks, and benefits was presented to the owner, and informed consent was duly acquired from all owners participating in the research.

## Consent

As the corresponding author, I hereby confirm that all authors have reviewed and approved the final version of this manuscript. We consent to its submission and potential publication in Journal of Equine Veterinary Sciences.

## Conflicts of Interest

The authors declare no conflicts of interest.

## Supporting Information

Additional supporting information can be found online in the Supporting Information section.

## Supporting information


**Supporting Information** Supporting Information includes Supporting Figures S1–S3 and Supporting Table S1, which provide representative flow cytometry plots and the CASA settings used for sperm motion analysis.

## Data Availability

Our data and materials would be available from the corresponding author upon reasonable request.
