# Supplementary material for: Effects of the Freezing Diluent Tonicity and Prefreezing Cooling Rates on Post‐Thaw Sperm Quality Markers in the Pure‐Bred Arabian Stallion
Source: Vet Med Int. 2026 Aug 13;2026:4438005. doi: 10.1155/vmi/4438005 (PMC13470873; doi:10.1155/vmi/4438005)
Supplement: Supplementary file 1 [file VMI-2026-4438005-s001.zip › SF2.docx]

Supplementary Figure 2. Flow cytometric images of inner mitochondrial membrane potential stained with Rhodamine 123/PI. The upper row graphs (from left to right) represent unstained sperm cells, Rhodamine single-stained sample, PI single-stained sample, respectively. The lower row graphs represent two-stained samples from different osmotic pressure groups, 330, 390, and 450, respectively.
