# Supplementary material for: Effects of the Freezing Diluent Tonicity and Prefreezing Cooling Rates on Post‐Thaw Sperm Quality Markers in the Pure‐Bred Arabian Stallion
Source: Vet Med Int. 2026 Aug 13;2026:4438005. doi: 10.1155/vmi/4438005 (PMC13470873; doi:10.1155/vmi/4438005)
Supplement: Supplementary file 1 [file VMI-2026-4438005-s001.zip › ST1.docx]

**Supplementary Table S1. CASA system settings.**

| **Category** | **Parameter** | **Value** |
| --- | --- | --- |
| **Sperm detection** | Minimum area | 8 pixels |
|  | Maximum area | 40 pixels |
|  | Mean area | 20 pixels |
| **Motility classification** | Motile minimum speed | 19.9 µm/s |
|  | Motile medium speed | 21.2 µm/s |
|  | Motile maximum speed | 250 µm/s |
| **Velocity thresholds** | VSL low | 5 µm/s |
|  | VSL high | 10 µm/s |
| **Linearity thresholds** | LIN low | 15% |
|  | LIN high | 30% |
| LIN: linearity; VSL: straight-line velocity. | | |
